# Unilateral cerebral cortical encephalitis (CCE) with positive anti-MOG antibodies: A case report

**DOI:** 10.1097/MD.0000000000026087

**Published:** 2021-05-21

**Authors:** Hongbing Nie, Haifeng Gao, Yongqiu Li, Yaoyao Shen

**Affiliations:** a Department of Neurology, Jiangxi Provincial People's Hospital Affiliated to Nanchang University, Nanchang, Jiangxi Province, China; b Department of Neurology, Tangshan Gongren Hospital, Tangshan, Hebei Province, China.

**Keywords:** cerebral cortical encephalitis, headache, myelin oligodendrocyte glycoprotein, seizures

## Abstract

**Rationale:**

Nowadays, myelin oligodendrocyte glycoprotein (MOG)-antibody-associated disease (MOGAD) is regarded as an independent inflammatory demyelinating disease. Here, we report a rare case of unilateral cerebral cortical encephalitis (CCE) with positive anti-MOG antibodies.

**Patient concerns:**

A 19-year-old woman was admitted to our hospital owing to acute onset fever and headache. Four days later, she experienced a focal seizure that progressed to generalized tonic-clonic seizures.

**Diagnosis:**

Brain magnetic resonance imaging (MRI) demonstrated cortical lesions in the left cerebral hemisphere on T2-weighted fluid-attenuated inversion recovery imaging. The patient was positive for anti-MOG antibodies in serum and diagnosed with anti-MOG antibody-associated unilateral CCE.

**Interventions:**

She was administrated with intravenous methylprednisolone followed by oral corticosteroids.

**Outcomes:**

On day 14 after admission, a repeat MRI revealed partial resolution of the initial abnormalities. The patient received a quick recovery without residual symptoms.

**Conclusions:**

Unilateral CCE with positive anti-MOG antibodies has emerged as a special clinical phenotype of MOGAD. It should be emphasized that the characteristic neuroradiological features of CCE would be an important clue to the correct diagnosis of MOGAD.

## 1 Introduction

Myelin oligodendrocyte glycoprotein (MOG), a protein exclusively expressed on the surface of oligodendrocytes and myelin sheaths in the central nervous system (CNS), is immunopathogenetically distinct from classic multiple sclerosis and AQP4-IgG-positive neuromyelitis optica spectrum disorders.^[1]^ Antibodies against MOG can be associated with a spectrum of clinical phenotypes, such as optic neuritis, myelitis, aseptic meningitis, encephalitis, and acute disseminated encephalomyelitis. However, unilateral cerebral cortical encephalitis (CCE) is a rare anti-MOG phenotype that was first described by Ogawa et al^[2]^ in 2017. The common symptoms of anti-MOG-associated unilateral CCE include seizures, headache, fever, and cortical symptoms. The characteristic finding on magnetic resonance imaging (MRI) is unilateral cortical hyperintensities on T2-weighted fluid-attenuated inversion recovery (T2-FLAIR) sequences.^[3]^ Recently, a new term “FLAMES” has been proposed to characterize the clinical and radiological syndrome in patients with unilateral cortical FLAIR-hyperintense lesions in anti-MOG-associated encephalitis with seizures.^[4]^ Here, we report a rare case of unilateral CCE with positive anti-MOG antibodies, which may expand the clinical phenotypes of MOG-antibody-associated disease (MOGAD).

## 2 Case presentation

**Figure 1:**
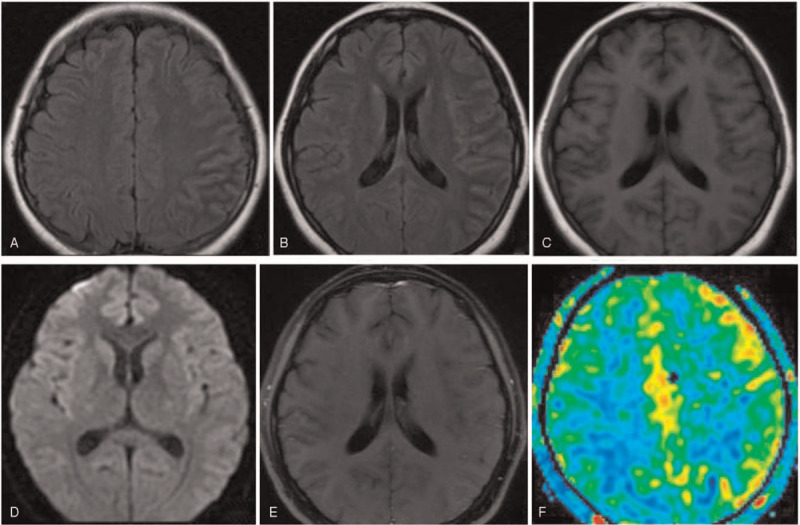
Brain MRI of our patient with anti-MOG-associated cerebral cortical encephalitis. On admission, axial T2-FLAIR images showed hyperintensity and swelling of the left cerebral cortex (A, B), without signal intensity changes on T1-weighted and diffusion-weighted images (C, D). Axial T1-weighted image post-gadolinium revealed no contrast enhancement (E). ASL image showed focal hyper-perfused areas involving the left cerebral cortex (F). ASL = arterial spin labelling, MRI = magnetic resonance imaging, T2-FLAIR = T2-weighted fluid-attenuated inversion recovery.

**Figure 2:**
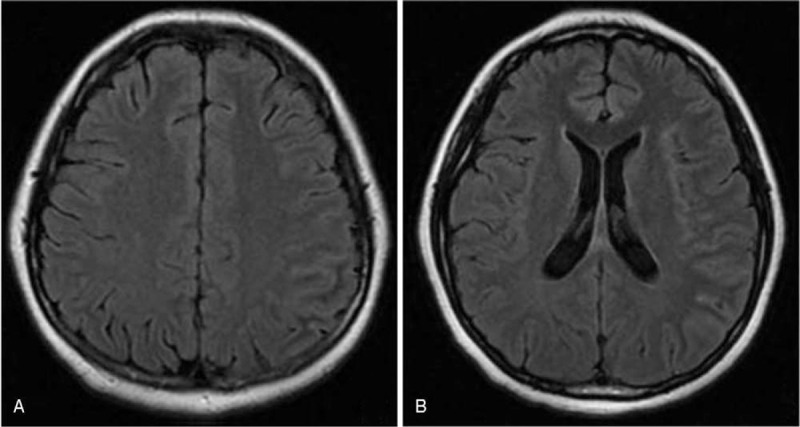
On day 14 after admission, axial T2-FLAIR images showed partial resolution of the left cerebral cortical lesions (A, B). T2-FLAIR = T2-weighted fluid-attenuated inversion recovery.

A 19-year-old woman was admitted to our hospital owing to paroxysmal headache and low-grade fever for 5 days. The headache was pulsatile, mainly in left frontoparietal lobe, accompanying with photophobia and fatigue. The body temperature was fluctuant, ranging from 37.3 to 38.5°C. Four days later, she suffered from a focal seizure affecting the right side of her face that progressed to generalized tonic-clonic seizures. On admission, her vital signs were a temperature of 38.3°C, a heart rate of 100 beats per minute, and blood pressure of 114/63 mmHg. She was alert and oriented. There were no meningeal irritation signs or focal neurologic deficits. Complete blood cell counts revealed leukocytosis 12.8 × 10^3^ cells/μL with segmented neutrophils 80.5%. No evidence of abnormality was found in other laboratory examinations, including biochemistry, tumor markers, thyroid hormones, C-reactive protein, and erythrocyte sedimentation rate. On day 2 after admission, brain MRI disclosed cortical lesions in the left cerebral hemisphere on T2-FLAIR imaging with slightly swelling (Fig. 1A, B). No signal intensity changes appeared on T1-weighted and diffusion-weighted images and there was no contrast enhancement after gadolinium injection (Fig. 1C-E). A perfusion image obtained by arterial spin labeling technique revealed focal hyperperfusion in the left cerebral hemisphere (Fig. 1F). An electroencephalogram recorded at the postepileptic stage was unremarkable. Cerebrospinal fluid (CSF) analysis showed increased cell count (46 cells/mm^3^, mononuclear cells dominant) and protein (0.54 g/L), with normal glucose and chloride. Oligoclonal bands were absent. Work-up for autoimmune encephalitis-related autoantibodies, including *N*-methyl-d-aspartate-receptor (NMDAR) antibodies, contactin associated protein 2 antibodies, leucine-rich glioma inactivated 1 antibodies, anti-voltage-gated potassium channel antibodies, anti-α-amino-3-hydroxy-5-methyl-isoxazolepropionic acid receptor antibodies, and anti-γ-aminobutyric acid-B receptor antibodies, were negative both in serum and CSF. Test for infectious pathogens (herpesviruses, *Mycobacterium tuberculosis*, HIV, syphilis, and lyme disease) were unremarkable. A cell-based assay detected the patient was positive for anti-MOG antibodies (1:512) and negative for anti-AQP4 in serum. She was administrated with high-dose glucocortieoid (methylprednisolone 1 g/day for 3 days) followed by gradually reduce to oral prednisolone. On day 14 after admission, a repeat MRI revealed partial resolution of the initial abnormalities (Fig. 2A, B). The patient received a quick recovery and was discharged without residual symptoms on day 14.

## 3 Discussion

Encephalitis is an important clinical phenotype of MOGAD. Anti-MOG-associated encephalitis frequently involves supratentorial deep white matter, cortical greyjuxtacotical white matter, pons, cerebellum, midbrain, medulla, and corpus callosum.^[5]^ However, anti-MOG-associated CCE is rarely reported.^[6]^ Previously, Budhram et al systematically reviewed 20 cases of anti-MOG-associated CCE from the literature.^[4]^ The most common symptom was seizures (85%), followed by headache (70%), fever (65%), and cortical symptoms (55%). The overwhelming majority of patients presented with at least 2 of these 4 symptoms. Among these patients, CCE was always limit to unilateral hemispheric cortex, but less bilateral. In comparison with unilateral CCE, anti-MOG antibody-associated bilateral medial frontal CCE presented with several characteristic clinical symptoms, such as paraparesis and lethargy.^[7]^ Moreover, 30% of patients reported adjacent sulcal hyperintensity on FLAIR and/or post-gadolinium leptomeningeal enhancement. Presently, unilateral cortical T2-FLAIR hyperintensities with epileptic seizure were regarded as a characteristic of anti-MOG-associated encephalitis.

Our case presented with headache, fever, and seizure, with slightly elevated CSF cells and protein. Together with cerebral cortical T2-FLAIR hyperintense lesions on brain MRI and high titer of anti-MOG antibody in serum, the clinical and neuroradiological features fulfill the diagnosis of anti-MOG-associated CCE. The relationship between MOG antibody and the unilateral CCE remains unclear. Brain biopsy studies of anti-MOG-associated CCE are seldom. Lymphocytic infiltration of both the subarachnoid space and brain parenchyma, as well as perivascular involvement, has been observed, but evidence of demyelination is largely absent.^[8]^ Besides, there is no elevation in CSF myelin basic protein despite extensive cortical involvement.^[8,9]^ Thus, whether anti-MOG antibodies are directly associated with unilateral CCE is controversial. It is worth noting that anti-MOG antibodies can also be detected in other autoantibody-associated encephalitis, such as anti-NMDAR encephalitis. It is speculated that the immune attack targeting myelin may involve NMDAR at the same time.^[10]^

CCE poses a broad differential diagnosis which includes seizure-induced brain MRI abnormalities, Rasmussen encephalitis (RE), Creutzfeldt-Jakob disease (CJD), and autoimmune encephalitis.^[11–14]^ Brain MRI abnormalities induced by seizure are usually localized in neocortex, subcortical white matter, corpus callosum, hippocampus, or basal ganglia, and often show restricted diffusion because of cytotoxic edema. RE is characterized by focal epilepsy, progressive hemiplegia, and cognitive decline with unilateral hemispheric focal cortical atrophy in the chronic stage, and is only partially responsive to corticosteroid. Sporadic CJD is a fatal progressive neurodegenerative disease and often presents with progressive dementia. The typical MRI appearance of CJD is increased diffusion-weighted image signal in the cerebral cortex and basal ganglia. The lesion distribution of autoimmune encephalitis is frequently located in the limbic lobe. Positive workup for those antibodies is critical to accurate etiology diagnosis.

In conclusion, unilateral CCE may be a special clinical phenotype of MOGAD. Anti-MOG-associated CCE often manifests as seizures, headache, fever, and cortical symptoms. The relationship between CCE and MOG antibodies is not well understood. More large-scale prospective studies need to be conducted for elucidating the robustness of the association.

## Author contributions

**Conceptualization:** Yaoyao Shen, Hongbing Nie.

**Data curation:** Hongbing Nie, Haifeng Gao, Yongqiu Li.

**Formal analysis:** Hongbing Nie, Haifeng Gao, Yongqiu Li.

**Methodology:** Hongbing Nie, Yaoyao Shen.

**Project administration:** Yaoyao Shen.

**Writing – original draft:** Yaoyao Shen.

**Writing – review & editing:** Hongbing Nie, Haifeng Gao, Yongqiu Li, Yaoyao Shen.
